# Study on the Correlation between Urinary Sodium and Potassium Excretion and Blood Pressure in Adult Hypertensive Inpatients of Different Sexes

**DOI:** 10.1155/2022/1854475

**Published:** 2022-06-30

**Authors:** Li Qin Duan, Xiao Yang Li, Qiong Li, Jin Fang Zhao, Li Zhao, Jun Zhang, Ze Hui Wang, Qing Hua Han

**Affiliations:** ^1^Department of Cardiology, The First Hospital of Shanxi Medical University, Taiyuan 030001, China; ^2^Shanxi Medical University, Taiyuan 030001, China; ^3^Department of Neurology, The First Hospital of Shanxi Medical University, Taiyuan 030001, China; ^4^Department of Statistics, Shanxi Medical University, Taiyuan 030001, China

## Abstract

**Objective:**

This study aims to understand the difference in the influence of urinary sodium and potassium excretion on blood pressure in patients of different sexes with hypertension by analyzing the relationship between urinary sodium and potassium excretion and blood pressure.

**Methods:**

In this cross-sectional study, 606 hospitalized patients with essential hypertension were recruited from 16 hospitals in the Shanxi Province between June 2018 and December 2019. These patients were grouped by sex, with 368 males and 238 females. Basic information and relevant serum biochemical indexes of patients in the two groups were recorded. The 24-hour urinary sodium and potassium excretion were measured, and 24-hour ambulatory blood pressure monitoring was performed simultaneously. This was done to analyze and compare the relationship between urinary sodium and urinary potassium excretion and blood pressure in adult hospitalized patients of different sexes with hypertension.

**Results:**

The 24-hour urinary sodium excretion in male patients with hypertension was significantly higher than that in female patients (*P* < 0.001). There was no significant difference in 24-hour urinary potassium excretion between male patients with hypertension and female patients. Spearman correlation analysis showed that 24-hour urinary sodium excretion was positively correlated with 24-hour SBP and nSBP in male patients (*P* < 0.05), while 24-hour urinary potassium excretion was negatively correlated with 24-hour SBP and nSBP in male patients (*P* < 0.05). The 24-hour urinary sodium in female patients was significantly positively correlated with 24-hour SBP, 24-hour DBP, SBP, dDBP, nSBP, and nDBP (*P* < 0.01). The 24-hour urinary potassium was significantly negatively correlated with nSBP (*P* < 0.05). Multiple stepwise linear regression showed that 24-hour urinary sodium excretion was still significantly positively correlated with 24-hour SBP and nSBP in male patients with hypertension after adjusting for various confounding factors.

**Conclusion:**

High urinary sodium and low urinary potassium excretion are closely related to elevated blood pressure in adult patients with hypertension, and there are sex differences.

## 1. Introduction

Hypertension is one of the most common chronic noncommunicable diseases worldwide and has become the most important risk factor for coronary heart disease, stroke, chronic kidney disease, and death [1, 2]. At present, the prevalence of hypertension among the Chinese population remains high and shows an increasing trend year by year [3, 4]. The etiology and pathogenesis of hypertension are complex. Genetic factors, environmental factors, dietary habits, gender factors, and other factors can affect the occurrence and development of hypertension [5, 6]. In dietary habits, high sodium intake is an important risk factor for hypertension, and previous evidence has revealed a linear correlation between sodium intake and blood pressure [7]. To date, several studies have demonstrated that high sodium intake is directly associated with an increased risk of cardiovascular morbidity and mortality [8, 9]. Globally, approximately 1.65 million cardiovascular deaths are attributed to excessive sodium intake each year [10]. The dietary structure of the Chinese population is generally characterized by high sodium (12–15 g/d) and low potassium intake (1.5–1.9 g/d) [11, 12]. In addition, previous studies have confirmed that the physiological structure differences between the sexes lead to differences in the regulation of blood pressure homeostasis at the tissue, cell, and molecular levels. Such differences lead to differences in the susceptibility, prevalence, and treatment response of patients with hypertension, depending on their gender. The animal study by Sampson AK et al. demonstrated that chronic low-dose infusion of AngII reduced MAP in female rats through AT2R-mediated effects and also in the absence of background AT1R blockade. Moreover, the mRNA expression of left ventricular AT2R, renal AT2R, and renal ACE2 in females is higher than that in males. After AngII infusion, the mRNA expressions of these RAS components also have differential changes, which may promote vasodilation in females and vasoconstriction in males [13]. Neugarten et al. demonstrated that both female sex and exogenous estrogen can increase iNOS (inducible nitric oxide synthase) levels and affect vasodilation [14]. McGuire et al. suggested that estrogens stimulate both endothelial nitric oxide synthase (eNOS) activity and NO production, and androgens positively regulate plasma renin activity (PRA), and estrogens antagonize this effect by increasing NO production [15]. Forte et al. also showed that under ambulatory conditions, whole-body NO biosynthesis was higher in healthy premenopausal women than in men. Differences in vascular endothelial NO production may contribute to differences in vascular function and susceptibility to arterial disease in men compared with women [16]. In addition, some studies have also found that the level of ET-1 in age-matched men is higher than that in women. However, women have enhanced ETB receptor function, and the effect of ETA may be reduced, in which ETB receptors produce NO through endothelial cells and renal tubular cells to cause vasodilation and urinary sodium excretion [17–19]. Therefore, ETB receptors provide a protective mechanism for elevated blood pressure, which is also one of the physiological mechanisms of lower blood pressure in women.

At present, although there are many studies at home and abroad on the relationship between urinary sodium, potassium excretion, and blood pressure, the relationship between these patients of different genders with hypertension is rare. This study attempted to analyze the relationship between urinary sodium and potassium excretion and blood pressure in adult patients with hypertension of different sexes in the Shanxi area and then to understand the difference in the influence of urinary sodium and potassium excretion on blood pressure in patients from this area; therefore, providing a new theoretical basis for the prevention and treatment of blood pressure.

## 2. Materials and Methods

### 2.1. Subjects

In this cross-sectional study, 606 hospitalized patients (368 males and 238 females), aged between 18 and 80 years, with essential hypertension were recruited from 16 hospitals in the Shanxi Province between June 2018 and December 2019. According to the scientific statement of the Chinese Medical Doctors Association on the diagnostic criteria and antihypertensive targets of hypertension in China, the diagnostic criteria for hypertension are systolic blood pressure ≥140 mmHg (1 mmHg = 0.133 kPa) or diastolic blood pressure ≥90 mmHg [20]. Patients with secondary hypertension, severe arrhythmia, congenital heart disease, valvular disease, long-term heavy use of diuretics, acute disease, pregnancy, connective tissue disease, and malignant tumors were excluded. All patients provided written informed consent. This research scheme was approved by the Ethics Committee of the First Hospital of Shanxi Medical University.

The oral antihypertensive drugs given to patients included calcium channel blockers (i.e., amlodipine besylate tablets, levamlodipine besylate tablets, nifedipine sustained-release tablets, nifedipine controlled-release tablets, benidipine, lacidipine, and felodipine sustained-release tablets), beta-blockers (i.e., bisoprolol, arolol, and metoprolol), ACEI or ARB (i.e., valsartan, candesartan, losartan, olmesartan, telmisartan, irbesartan, benazepril, enalapril, and perindopril), and single-piece compound preparation (valsartan ammonia clodipine tablets, etc.).

### 2.2. Research Methods

The general clinical data, including gender, age, height, weight, and history of hypertension, were collected. Body mass index (BMI) = weight (kg)/height (m)^2^ were calculated. Fasting blood samples were taken during the hospital stay. Biochemical indicators, including fasting blood glucose (FBG), total cholesterol (TC), triglyceride (TG), low-density lipoprotein cholesterol (LDL-C), and high-density lipoprotein cholesterol (HDL-C), were collected using an automated analyzer (DXC800, Beckmann Coulter).

Collection of 24-hour urine sodium and potassium: a 24-hour urine sample was collected from all subjects on the second day of hospitalization. Urine collection instructions and a unified collection container were distributed to the participants, explaining in detail the precautions for urine collection. Patients maintained a regular diet on the day of urine collection and avoided strenuous exercise to reduce sweating. The 24-hour urine samples were collected for all subjects, and the starting and ending time of urine collection was recorded to determine the total urine volume collected for 24 hours. Any urine retention time less than 24 hours or urine loss or 24-hour urine volume <500 mL were excluded. All urine samples were labeled and stored for measurement. The concentration of urinary sodium and potassium was determined by an ion-selective electrode method, and the excretion of urinary sodium and potassium was calculated at 24 hours.

Collection of patients' ambulatory blood pressure data: 24-hour ambulatory blood pressure monitoring (24-hour ABPM) was performed using a domestic HingmedABP-03B noninvasive portable ambulatory blood pressure analyzer to comprehensively monitor diurnal changes in blood pressure with high repeatability. The monitoring frequency was set as follows: in the daytime (6:00–2200), the blood pressure was automatically inflated to measure blood pressure once every 15 minutes, and at night (22:00–6:00 the next day), the blood pressure was automatically inflated to measure blood pressure once every 30 minutes. If the effective blood pressure reading reached more than 70% of the measured times, the total monitoring time was 24 hours. After 24 hours, blood pressure data were transmitted to the ambulatory blood pressure management software. After excluding invalid blood pressure data, the average systolic and diastolic blood pressure of the patients at 24 hours, daytime, and nighttime, were analyzed and recorded. The following indicators were used for ambulatory blood pressure elevation: 24-hour threshold ≥130/80 mmHg, daytime threshold ≥135/85 mmHg, and nighttime threshold ≥120/70 mmHg.

### 2.3. Statistical Analysis

Nonparametric continuous variables were presented as the median (interquartile range) and were compared using the nonparametric Wilcoxon rank-sum test for two independent samples. Categorical variables were expressed in percentages and compared using the Chi-square test. Rank correlation analysis was used to study the relationship between 24-hour urinary sodium and urinary potassium excretion and 24-hour ambulatory blood pressure parameters in men and women. Multiple stepwise linear regression was used, and important confounding factors were included in the model for adjustment. *P* values less than 0.05 were considered statistically significant. The relationship between 24-hour urinary sodium, urinary potassium excretion, and 24-hour ambulatory blood pressure parameters in male and female patients was analyzed. *P* values less than 0.05 were considered statistically significant. Analyses were carried out using SPSS software (version 26.0).

## 3. Results

### 3.1. General Data of Patients in Each Group

The 24-hour urinary sodium excretion in male patients [176.57 (144.00∼215.95) mmol] was higher than that in female patients [164.00 (129.75∼198.50) mmol], and the difference was significant (*Z* = −3.643, *P* < 0.001). There was no significant difference in 24-hour urinary potassium excretion between male patients [39.93 (30.97–52.98) mmol] and female patients [40.16 (27.81–55.46) mmol] (*Z* = −0.375, *P*=0.708).

We analyzed the general clinical data of the selected subjects and found that there was no statistical significance between the history of diabetes and the history of hyperlipidemia in both males and females. However, there were significant differences in age and BMI (Table 1).

### 3.2. Comparison of 24 Hour Ambulatory Blood Pressure Parameters in Male and Female Patients with Hypertension

The 24-hour ambulatory blood pressure parameters of the male and female hypertensive patients were compared. The results showed that the 24 hSBP, dSBP, and nSBP of the male group were not significantly different from those of the female group (*P* > 0.05), but the 24hDBP, dDBP, and nDBP of the male group were significantly higher than those of the female group (*P* < 0.001) (Table 2).

### 3.3. Rank Correlation Analysis of 24-Hour Urinary Sodium and Urinary Potassium Excretion and Blood Pressure in Patients of Different Genders

Spearman correlation analysis in the male group showed that 24-hour urinary sodium excretion was significantly positively correlated with 24-hour SBP and nSBP (*P* < 0.05). The 24-hour urinary potassium excretion was significantly negatively correlated with 24-hour SBP and nSBP (*P* < 0.05). Spearman correlation analysis in the female group showed that 24-hour urinary sodium was significantly positively correlated with 24-hour SBP, 24-hour DBP, dSBP, dDBP, nSBP, and nDBP (*P* < 0.01), while 24-hour urinary potassium was significantly negatively correlated with nSBP (*P* < 0.05) (Table 3).

### 3.4. Multiple Stepwise Linear Regression between 24-Hour Urinary Sodium and Potassium Excretion and 24-Hour Ambulatory Blood Pressure Parameters

We established a model using multiple stepwise linear regression to analyze the correlation between 24-hour urinary sodium and potassium excretion and 24-hour SBP, 24-hour DBP, dSBP, dDBP, nSBP, and nDBP of the different sexes. After adjusting for age, BMI, history of diabetes, and history of hyperlipidemia, the results showed that 24-hour urinary sodium excretion was positively correlated with 24-hour SBP and nSBP in male patients with hypertension (*P* < 0.05), and 24-hour urinary potassium excretion was negatively correlated with 24-hour SBP, dSBP, and nSBP in male patients with hypertension (*P* < 0.05). The 24-hour urinary sodium was still significantly positively correlated with 24-hour SBP, 24-hour DBP, dSBP, dDBP, nSBP, and nDBP in female patients with hypertension (*P* < 0.01), while 24-hour urinary potassium was significantly negatively correlated with 24-hour SBP, dSBP, and nSBP (*P* < 0.05) (Table 4).

## 4. Discussion and Analysis

The prevention of cardiovascular and cerebrovascular diseases is an important public health strategy to improve national health and further increase life expectancy. Among cardiovascular and cerebrovascular diseases, hypertension, in particular, has a high incidence worldwide, so the prevention and treatment of hypertension is a top priority. In the past 20 years, China has carried out several national sample surveys on people over 18, and the results of three sample surveys conducted from 2002 to 2015 show that the prevalence of hypertension among the overall population is on the rise [4, 21]. In China, people are generally sensitive to sodium, and sodium plays an important pathophysiological role in the development of blood pressure and hypertension [22]. Epidemiological studies (such as the INTERSALT study) [7] have shown a significant positive correlation between urinary sodium and individual blood pressure. A 100 mmol reduction in daily sodium intake was associated with an average reduction of 9 mmHg in systolic blood pressure and 4.5 mmHg in diastolic blood pressure in adults aged 25–55. There is a close relationship between excessive sodium intake and increased cardiovascular and cerebrovascular events [7, 23]. In addition, the study of Mente et al. showed that potassium has a lowering effect on blood pressure, and potassium intake is negatively correlated with the risk of death, cardiovascular events, and blood pressure [24–26]. Strazzullo et al. observed the highest blood pressure in individuals with the highest sodium excretion and the lowest potassium excretion [27].

Sodium and potassium are the most abundant cations, both extracellular and intracellular, and they are inextricably linked to maintaining the homeostasis of the organism. High sodium intake directly causes sodium and water retention and increases circulating blood volume, resulting in an increased return to the heart and cardiac output, resulting in increased blood pressure. Increased sodium levels will amplify the baroreflex of sympathetic nerve activity and lead to a strong sympathetic nervous response and eventually an increase in blood pressure [28]. High sodium intake will continuously activate the renin-angiotensin-aldosterone system, specifically, inhibit nitric oxide-mediated vascular dilatation, cause endothelial dysfunction, and promote the proliferation of vascular smooth muscle cells and fibrosis of cardiomyocytes, thereby affecting blood pressure [29, 30]. This is the main mechanism by which high sodium affects blood pressure and is an early stage in the progression of cardiovascular disease. Potassium may play a role in protecting endothelial function and improving arterial compliance to lower blood pressure. These roles may include stimulation of natriuretic peptides, the release of carbon monoxide, and the improvement of endothelial function. In addition, endothelial hyperpolarization and cytoplasmic smooth muscle calcium were decreased by the stimulation of sodium and potassium pumps and plasma membrane potassium channels or reduced the activity of the sympathetic nervous system, which relaxed the vascular smooth muscle [31–33].

The sodium and potassium in the human body are mainly excreted by the kidneys, and the intake is approximately equal to the excretion in urine when the diet is stable. Therefore, the intake of the body is estimated by the excretion of urinary sodium and potassium in 24 hours when the diet is stable. In this study, 24-hour urine samples were collected to analyze the relationship between urinary sodium and urinary potassium excretion and blood pressure for the first time. The results showed that the average salt intake of adult patients with hypertension in Shanxi Province was 10.65 g/d. In males, the intake was 11.15 g/d, and in females, it was 9.79 g/d. This is significantly higher than the World Health Organization's recommendation of no more than 5 g of salt per person per day (the Dietary Guidelines for Chinese Residents [2016] recommend no more than 6 g of salt per day for adults). The average potassium intake per capita was 1.72 g/L, the average potassium intake for males was 1.77 g/d, and the average potassium intake for females was 1.63 g/d. Both are lower than the amount of 2 g recommended in the Dietary Guidelines for Chinese Residents (2016). The 24-hour ambulatory blood pressure monitoring, which was used in the present study, has been widely used in clinical practice. It provides more comprehensive and real-time blood pressure information and is able to identify different types of hypertension (e.g., masked hypertension and nocturnal hypertension). The 24-hour ambulatory blood pressure monitoring records blood pressure day and night to obtain the 24-hour average blood pressure. It is a relatively objective and accurate detection method for evaluating the blood pressure levels with strong reliability and repeatability. In addition, it is possible to analyze the circadian rhythm of blood pressure and the trend of blood pressure changes, which may guide treatment plans and evaluate antihypertensive efficacy and reduce the risk of target organ damage. The study of this research group showed that in both male and female patients with essential hypertension, 24-hour urinary sodium excretion was significantly positively correlated with increased blood pressure, while 24-hour urinary potassium excretion was significantly negatively correlated with increased blood pressure. After adjusting for confounding factors such as age, BMI, history of diabetes, and history of hyperlipidemia, multiple linear stepwise regression analysis showed that the regression relationship between 24-hour urinary sodium and potassium excretion and 24-hour ambulatory blood pressure parameters was still statistically significant. This correlation was more significant in female patients with hypertension.

A previous study has confirmed that being female is also one of the important factors affecting the mechanism of high sodium and low potassium promoting hypertension [34]. Prospective antihypertensive drug studies have shown that when using angiotensin receptor antagonists and hydrochlorothiazide as antihypertensive agents, women are more sensitive to the drugs and have better control than men [35–37]. Therefore, gender differences should be considered in the prevention of hypertension, drug selection, and dietary intervention. Our study found that the correlation between 24-hour urinary sodium, urinary potassium excretion, and blood pressure was more significant in female patients, which was consistent with previous research results. The mechanism for this sex difference may be attributed to aldosterone-mediated vascular endothelial dysfunction and the possibility of genetic predisposition. Shukri et al. [38] suggested that women might respond to increased dietary sodium by maintaining or increasing aldosterone production. The levels of aldosterone and the responsiveness to angiotensin-converting enzyme and potassium were significantly higher in women than in men, and angiotensin II stimulated higher production of aldosterone in women than in men. The activation of the endothelial mineralocorticoid receptor expression by aldosterone leads to endothelial dysfunction, leading to increased blood pressure [34, 38], which reasonably explains the gender differences in hypertension epidemiology. These studies suggest that more attention needs to be paid to RAAS levels when treating women with hypertension. Diuretics and mineralocorticoid receptor antagonists may be more effective in blood pressure management in women with hypertension.

High sodium and low potassium intake is a controllable factor in blood pressure development in adult patients with hypertension of different sexes. In the prevention and treatment of hypertension in the future, we should vigorously publicize the health significance of a balanced diet of sodium and potassium, carry out reasonable diet management according to different sexes, and develop individualized antihypertensive programs. This study first analyzed the relationship between urinary sodium, potassium excretion, and blood pressure in different gendered patients with hypertension, which is innovative in guiding blood pressure management in these patients. Moreover, the sample size of the study is large, the subjects are regionally representative, and the results of the study have great reference value. However, electrolyte excretion is highly variable in daily life and has many influencing factors. In this study, 24-hour urine samples were used in a single day, which is a deficiency of this study. With sufficient conditions, we can collect urine for 24 hours for several days to obtain more accurate data.

## 5. Conclusion

High urinary sodium and low urinary potassium excretion are closely related to elevated blood pressure in adult patients with hypertension in Shanxi, and there are gender differences. There was a stronger positive correlation between high urinary sodium excretion and blood pressure in women than in men. Low urinary potassium excretion was significantly negatively correlated with 24-hour mean systolic blood pressure and nighttime systolic blood pressure in patients with hypertension. Gender differences should be considered in the prevention, treatment, and dietary intervention of patients with hypertension.

## Figures and Tables

**Table 1:** General data of patients in each group.

Items		Male (*n* = 368)	Female (*n* = 238)	*Z*/*χ*^2^	*P*
Age (y)		56.75 (46.33∼65.47)	59.47 (52.77∼67.65)	−3.455	<0.001
Weight (kg)		76.00 (70.00∼84.38)	65.00 (60.00∼70.00)	−12.473	<0.001
Height (cm)		172.00 (168.00∼175.00)	160.00 (156.00∼163.00)	−18.208	<0.001
Body mass index ((kg/m^2^)		25.76 (24.22∼28.09)	25.24 (23.29∼27.90)	−2.067	0.039
24-hour urinary sodium (mmol/L)		176.57 (144.00∼215.95)	164.00 (129.75∼198.50)	−3.643	<0.001
24-hour urinary potassium (mmol/L)		39.93 (30.97∼52.98)	40.16 (27.81∼55.46)	−0.375	0.708
Diabetes	Yes	71 (18.7%)	37 (14.9%)	1.386	0.239
	No	297 (81.3%)	201 (85.1%)		
Hyperlipidemia	Yes	176 (47.0%)	108 (45.8%)	0.348	0.555
	No	192 (53.0%)	130 (54.2%)		

**Table 2:** Comparison of 24-hour ambulatory blood pressure parameters in male and female patients with hypertension.

Items	Male (*n* = 368)	Female (*n* = 238)	*Z*	*P*
24 hSBP	135.00 (126.00∼146.00)	133.00 (123.00∼144.00)	−1.634	0.102
24 hDBP	83.00 (76.00∼90.00)	79.00 (70.00∼85.000	−5.176	<0.001
dSBP	137.50 (128.00∼148.00)	135.50 (124.75∼146.00)	−1.856	0.063
dDBP	84.00 (77.00∼93.00)	79.00 (71.00∼87.25)	−5.168	<0.001
nSBP	128.00 (119.00∼141.00)	128.00 (116.75∼139.25)	−0.815	0.415
nDBP	79.00 (71.25∼88.00)	75.00 (68.00∼82.00)	−4.518	<0.001

*Note.* 24hSBP: 24-hour systolic blood pressure; 24hDBP: 24-hour diastolic blood pressure; dSBP: Daytime systolic blood pressure; dDBP: Daytime diastolic blood pressure; nSBP: Nighttime systolic blood pressure; nDBP: Nighttime diastolic blood pressure.

**Table 3:** Rank correlation analysis of 24-hour urinary sodium and urinary potassium excretion and blood pressure in patients of different genders.

Gender	Items	24-hour urinary sodium		24-hour urinary potassium	
		Correlation coefficient	*P*	Correlation coefficient	*P*
Male	24 hSBP	0.114	0.029	−0.115	0.028
	24 hDBP	0.056	0.285	0.001	0.992
	dSBP	0.091	0.080	−0.102	0.051
	dDBP	0.048	0.360	−0.010	0.845
	nSBP	0.130	0.013	−0.113	0.030
	nDBP	0.086	0.099	−0.011	0.832

Female	24 hSBP	0.299	<0.001	−0.100	0.124
	24 hDBP	0.239	<0.001	0.024	0.712
	dSBP	0.288	<0.001	−0.080	0.217
	dDBP	0.229	<0.001	0.053	0.417
	nSBP	0.271	<0.001	−0.147a	0.023
	nDBP	0.187	0.004	−0.017	0.790

**Table 4:** Multiple stepwise linear regression between 24-hour urinary sodium and potassium excretion and 24-hour ambulatory blood pressure parameters.

Male	24 hSBP							
	Unadjusted				After adjustment			
	b	S.E	*t*	*P*	b	S.E	*t*	*P*
24-hour urinary sodium	0.028	0.013	2.177	*P*=0.030	0.028	0.013	2.190	*P*=0.029
24-hour urinary potassium	−0.129	0.041	−3.188	*P*=0.002	−0.131	0.040	-3.232	*P*=0.001

	dSBP							
	Unadjusted				After adjustment			
	b	S.E	*t*	*P*	b	S.E	*t*	*P*
24-hour urinary potassium	−0.110	0.042	−2.594	*P*=0.010	−0.111	0.042	−2.650	*P*=0.008

	nSBP							
	Unadjusted				After adjustment			
	B	S.E	t	*P*	B	S.E	t	*P*
24-hour urinary sodium	0.035	0.014	2.506	*P*=0.013	0.035	0.014	2.506	*P*=0.013
24-hour urinary potassium	−0.127	0.044	−2.878	*P*=0.004	−0.127	0.044	−2.878	*P*=0.004

Female	24 hSBP							
	Unadjusted				After adjustment			
	b	S.E	*t*	*P*	b	S.E	*t*	*P*
24-hour urinary sodium	0.127	0.019	6.668	*P* < 0.001	0.123	0.019	6.480	*P* < 0.001
24-hour urinary potassium	−0.162	0.049	−3.288	*P*=0.001	−0.161	0.049	−3.285	*P*=0.001

	24 hDBP							
	Unadjusted				After adjustment			
	b	S.E	*t*	*P*	b	S.E	*t*	*P*
24-hour urinary sodium	0.051	0.016	3.238	*P*=0.001	0.052	0.013	3.832	*P* < 0.001

	dSBP							
	Unadjusted				After adjustment			
	b	S.E	*t*	*P*	b	S.E	*t*	*P*
24-hour urinary sodium	0.125	0.020	6.174	*P* < 0.001	0.121	0.020	5.994	*P* < 0.001
24-hour urinary potassium	−0.141	0.052	−2.702	*P*=0.007	−0.140	0.052	−2.690	*P*=0.008

	dDBP							
	Unadjusted				After adjustment			
	b	S.E	*t*	*P*	b	S.E	*t*	*P*
24-hour urinary sodium	0.059	0.015	4.041	*P* < 0.001	0.060	0.013	4.590	*P* < 0.001

	nSBP							
	Unadjusted				After adjustment			
	b	S.E	*t*	*P*	b	S.E	*t*	*P*
24-hour urinary sodium	0.125	0.021	6.049	*P* < 0.001	0.119	0.020	5.838	*P* < 0.001
24-hour urinary potassium	−0.180	0.053	−3.372	*P*=0.001	−0.177	0.052	−3.384	*P*=0.001

	nDBP							
	Unadjusted				After adjustment			
	b	S.E	*t*	*P*	b	S.E	*t*	*P*
24-hour urinary sodium	0.050	0.013	3.898	*P* < 0.001	0.049	0.011	4.279	*P* < 0.001

*Note.* The following variables were adjusted: age, BMI, history of diabetes, and history of hyperlipidemia.

## Data Availability

The datasets used and/or analysed during the current study are available from the corresponding author upon request.
